# The National Hotel Disease

**Published:** 1857-09

**Authors:** 


### HALL'S JOURNAL OF HEALTH.

OUR LEGITIMATE SCOPE IS ALMOST BOUNDLESS: FOR WHATEVER BEGETS PLEASURABLE AND HARMLESS FEELINGS, PROMOTES HEALTH; AND WHATEVER INDUCES DISAGREEABLE SENSATIONS, ENGENDERS DISEASE.

VOL. IV.] SEPTEMBER, 1857. [NO. IX.

WE aim to show how disease may be avoided, and that it is best, when sickness comes, to take no medicine without consulting an educated physician.

### THE NATIONAL HOTEL DISEASE.

HAVING been strongly and repeatedly urged by a lady sufferer at the National Hotel about the middle of March, and who came under my care during a relapse in New York, about the first of May, to publish my views of the malady, I have thought it might be of some service possibly to do so, as I have been in a position to obtain information not only from facts coming under my own observation, but from scientific men on the spot, who were personally familiar with the whole history of things from the beginning up to the present time.

Having lived in the midst of those malarial districts of the South, for a great portion of my life, opportunities have been afforded for becoming familiar with diseases of that nature and their various symptoms. My opinion is, that bad air, malaria will account for all the phenomena observed, and that no other supposition can.

Some five or six years since, diarrhœa was very prevalent at that hotel.

One gentleman was seized at the National last October.

An eminent physician at Washington says: "The first case of the diarrhœa I met with at the National was in the latter part of January."

It cannot be denied that several persons who never ate anything at that house were attacked by the disease. There were many who took all their meals there through the whole season, and remained well.

It is not in proof that there were any cases in the neighborhood. A wide street bounds the house on three sides.

Ladies who spent all their time in the building, suffered less than men who were absent a good part of the time, thus eating at the house less often than the women.

There was every grade of intensity in the symptoms in different persons.

The symptoms themselves were much varied. The attacks usually came on early in the morning and suddenly, with a copious and repeated discharge from the bowels of a light, colored, frothy, sourish fluid, of custard consistency, without pain, griping, straining, tenderness, chilliness, or fever.

In most cases, the stomach was not affected at all in the beginning, and the appetite would remain good.

If the disease went on for a few days, the stomach became irritated, and the food only was thrown up, especially if the bowels had been checked by opiates and astringents.

Sometimes very violent pains would be felt in the bowels, seemingly due to spasm and flatulence.

At a later period, inflammation, soreness, tenderness, and fever, would manifest themselves, with loss of appetite, great thirst, and in a very few cases, cramps.

A copious rash appeared in a gouty patient.

There were few, if any, cases of bloody or mucous stools.

The best results generally followed the use of absorbents and antacids, such as charcoal and magnesia.

The best diet seemed to be rice, farina, starch, and the like.

Quiet and confinement were less beneficial than courageously keeping out and about.

Relapses most frequently occurred from imprudence in eating, especially meat.

The stomach was only secondarily affected, and sometimes not at all.

One man would eat a single meal in the house, leave it, be taken sick, and remain so for weeks; another would eat and live in the house for days and weeks, and escape altogether.

Several persons who remained in the house and procured their own food and water, did not escape an attack.

Many parts of the house were filled with a most offensive odor, some parts were comparatively exempt.

No practical chemist has critically examined a body dead from the National Hotel disease, and declared under his own name that there was any trace of a mineral poison. Examinations of the dead body have been critically made, without the discovery of arsenic or of any other mineral poison, as in the case of Mr. Petriken, of Harrisburg, Pa. In epidemics from bad air, this bad air including miasma, that is, decaying vegetation, as well as malaria in general, such as chills and fever, fever and ague, diarrhœa, dysentery, cholera, yellow fever, and the like, half the people escape altogether. If mineral poison is in the food on the table in sufficient quantities to affect a single person decidedly, such effects will be observed within a very few hours, and not one in ten can possibly escape altogether.

In large doses, arsenic kills in five or six hours, before inflammation takes place.

In moderate doses, there is inflammation and irritation, of the bowels, and death occurs in three or four days.

In a third class of cases, there is a nervous irritation causing a kind of palsy or lockjaw, hysterics, mania, and the like. If death does not take place in a few hours from arsenic, there is inflammation of the stomach, as well when it is applied to a fresh wound as when in the stomach.

For a hundred persons to sit down to a table and partake of food poisoned with arsenic, causing death in some instances or severe sickness within a few hours, and yet the greater number to escape, is a chemical and physiological impossibility.

Heat destroys the effects of malaria as to the human body by rarifying it, and carrying it above the breathing point. Cold brings it nearer the floor or earth, and in a more condensed and virulent form. Hence women sitting in warmer rooms with the larger supplies of air from without through window crevices, suffered less than men who were in colder rooms with a supply of new air from frequently opening doors from the passages in the hotel, the outer doors being kept closed.

Mineral poisons affect the stomach almost instantly, if in large doses, causing fearful vomitings or excessive burning or pains in throat, stomach, or bowels.

A person entering a house in a tired and wearied condition, and breathing a concentrated malaria while eating a single meal, and then leaving the house, may very easily imbibe a fatal disease in that short time, from the operation of two causes—his debilitated state, and the empty stomach drinking in the poison from every swallow of saliva, until he eats, and then the vigor of the system being expended on the digestion of the food in the stomach, leaves other parts of the system proportionably weak and unprotected against disease-engendering agencies.

Anomalous secondary symptoms and phenomena would attend the disease, from the fact of the extraordinary combination of simple miasma, with the more general malaria, and the extraordinary concentration of these gases, from sewers, cesspools, kitchen offal, noisome cellars, and the heat of cooking, washing, and ironing in the lower rooms of the building, not very accessible to the outdoor air.

It is believed that not a single statement has been made in the preceding article that cannot be literally substantiated in a court of justice by the testimony of personal observation of the facts of the case—the testimony of scientific men, favorably known to fame. The aim has been to write a reliable paper, hoping that competent minds abroad, as well as at home, may be able to deduce therefrom conclusions which will shield the National from a warrantable suspicion that there could exist, in its whole extent, a heart so base as to attempt the death of the President elect, all regardless, too, of the certain fatal consequences to hundreds of helpless women and innocent children, and unoffending fellow citizens.

The above was written by us for the *New York Herald*, about the first of June last, and copied from that paper by the "States" of Washington, at a time when our most learned city contemporary wrote: "The attempt to ascribe its origin to Miasm is everywhere justly ignored. We have never doubted that poison in food or drink was the source of all the disease and death which has resulted." Two months later, after a fuller investigation of authenticated facts, this writer declares his "belief that the source of the epidemic at the National Hotel at Washington, was solely a poisonous atmosphere. This foul air we regard as the *one common cause* which exposed (?) all who inhaled it to a predisposition to the malady, which itself was modified in individual cases by previous health, and developed with greater or less promptness and severity, by excesses or indiscretions in diet, drinks, exposure," &c.

Our Fifth Avenue Dissector, in his fearless, slap-dash style, says: "We have been amused at the solemn announcement of the medical committee appointed to pronounce this awful event —the secret of miasm. That this epidemic should have produced the set of symptoms it did without a specific or *material* poison acting on the stomach and its appendages, *is absurd.* Arsenic *mechanically diffused* from the decayed rats, and slowly acting on the stomach, is sufficient to account for all the symptoms."

Dr. Dixon is not afraid to do anything, not unbecoming— even to make an acknowledgment that he was mistaken. By the way, let every untidy housekeeper in the land send twenty-five cents to Sherman & Co., No. 1 Vesey street, New York, for the Scalpel of *July*, and read with wholesome fear, the effects of filthy housekeeping, page 116.

A medical writer in Cincinnati dogmatizes thus: "We have no doubt that every one was poisoned with arsenic. Any one either in or out of the medical profession, who will deny these facts, will at once prove to the world that he knows nothing of the action of this poison upon the human structure."

The New York Academy of Medicine, after a patient collection of evidence from the most reliable sources at Washington and elsewhere, declares, "that no known poisonous article from either kingdom of nature, would have produced all the group of symptoms which so uniformly characterize all the cases, and certainly not without involving the stomach itself in more serious mischief than is alleged to have been present in any case."

Dr. J. C. Hall, of Washington, who had a larger number of cases in his care than any other, himself one of the ablest physicians in the country, and one of the brightest ornaments of the medical profession, expresses himself in the most decided terms, that bad air was the cause, and the sufficient cause, of the National Hotel endemic.

On the other hand, a correspondent of so staid a paper as the *New York Observer*, writes, May 9, from Washington: "The exhalation theory is full of absurdities." The only reason given is, "One of my own family is certain he was poisoned."

These conflicting opinions are presented, not because we have any doubt that bad air was the sole cause, but to show that bad air alone can have an effect on the system sufficiently violent to induce unintelligent persons to believe them to be of arsenical origin. The practical point is this, let every reader hate dirt of every description, in atoms, as well as in tons, with a perfect hatred. The National Hotel disease is one of the most impressive lessons ever given as to the deadly nature of foul air—of filthy housekeeping. There was death in the pot, in the olden time, and death in it now every day; but foul cellars, back yards, kitchens, and dormitories, are the unsuspected causes of many a childless household.

